# Association between cardiometabolic index and testosterone levels in adult men: NHANES 2011–2016

**DOI:** 10.1371/journal.pone.0306401

**Published:** 2024-08-28

**Authors:** Xuanchun Huang, Lanshuo Hu, Shiyi Tao, Tiantian Xue, Jun Li, Xuejiao Wang

**Affiliations:** 1 Guang’anmen Hospital, China Academy of Traditional Chinese Medicine, Beijing, China; 2 Xiyuan Hospital, China Academy of Traditional Chinese Medicine, Beijing, China; Tehran University of Medical Sciences, ISLAMIC REPUBLIC OF IRAN

## Abstract

**Objective:**

Exploring the relationship between the cardiometabolic index (CMI) and serum testosterone levels as well as testosterone deficiency in American adult males. Additionally, comparing the diagnostic value of the CMI with several common obesity and metabolism indices for identifying testosterone deficiency.

**Methods:**

This cross-sectional study was conducted using data from the National Health and Nutrition Examination Survey (NHANES) from 2011 to 2016. Serum testosterone levels and testosterone deficiency were used as dependent variables, with the cardiometabolic index as the independent variable. Multivariable regression was used to assess the relationship between the independent and dependent variables, while subgroup analyses were performed to ensure the stability of the results. Smooth curve fitting was utilized to evaluate the nonlinear relationship between the CMI and testosterone levels. Receiver operating characteristic curves (ROC) were plotted for several obesity and metabolism prediction indices and the area under the curve was calculated to compare the specificity and sensitivity of each diagnostic index in the diagnosis of testosterone deficiency.

**Results:**

Among 3541 adult male participants, CMI is negatively associated with serum testosterone levels and positively associated with testosterone deficiency. In the fully adjusted model, for every unit increase in CMI, serum testosterone decreased by 14.89 ng/dl. Comparing the highest quartile to the lowest quartile of CMI, each unit increase in CMI, serum testosterone decreased by 98.58 ng/dl. Furthermore, each unit increase in CMI was associated with a 16% increase in incidence of testosterone deficiency. By plotting the ROC curves, we found that the AUCs for Lipid Accumulation Product (LAP), Body Mass Index (BMI), Weight Adjusted Waist Index (WWI), CMI, Visceral Adiposity Index (VAI) and Triglyceride glucose index (TyG) were 0.73, 0.72, 0.71, 0.69, 0.66, and 0.66 respectively.

**Conclusion:**

Elevated levels of CMI are associated with lower testosterone levels and an increased risk of testosterone deficiency. The predictive value of the LAP was superior to that of CMI, while the predictive value of CMI was higher than VAI and TyG.

## 1 Introduction

Testosterone is a crucial hormone for maintaining male secondary sexual characteristics and overall health [1]. In recent years, the significance of testosterone has received widespread attention. Studies have shown that testosterone deficiency is related to sexual dysfunction and decreased sperm quality, and it is also associated with the occurrence and development of cardiovascular diseases such as coronary heart disease, stroke, and hypertension [2–4]. Additionally, testosterone deficiency plays an important role in the onset and exacerbation of metabolic diseases like diabetes, obesity, and dyslipidemia [5, 6]. Therefore, identifying an index that can predict testosterone levels is of significant clinical importance for preventing testosterone deficiency-induced physical and mental diseases and for improving patients’ quality of life.

Recent studies have shown that patients with obesity and abnormal lipid metabolism have lower testosterone levels, and once obesity or dyslipidemia was being treated, testosterone levels can be significantly increased. These pieces of evidence suggest that testosterone levels are influenced by metabolic status and the degree of obesity [7–12]. This may be related to the fact that obese patients with lipid metabolism disorders are in a state of insulin resistance and hyperfunction of adipocytes, which leads to a chronic low-grade inflammatory state that inhibits the secretion of testosterone [13, 14]. Therefore, the cardiometabolic index (CMI) proposed by Ichiro Wakabayash in 2015 seems to perfectly assess the effect of lipids and obesity on testosterone [15]. Currently, this index, which combines lipid metabolism and body measurement indicators, effectively predicts the risk of diabetes, coronary heart disease, and metabolic syndrome due to obesity and dyslipidemia [16, 17]. It is considered a powerful and reliable indicator widely used in clinical research. Given the CMI’s evaluative value in lipid metabolism and obesity, we are confident that it has the potential to be developed as an effective predictor of testosterone levels in men.

In the past, researchers have used indicators such as BMI, WWI, VAI, LAP, and TyG to predict testosterone levels [18–21], and they found a negative relationship between these indicators and testosterone. However, these indicators either failed to integrate physical metrics with metabolic conditions, or their calculations were too complex, making it inconvenient and ineffective for clinicians to assess the impact of obesity and metabolism on testosterone levels. Therefore, exploring the relationship between the CMI and serum testosterone levels holds certain clinical significance. In order to more accurately understand the relationship between the CMI and serum testosterone, this study analyzes data from the National Health and Nutrition Examination Survey (NHANES). Additionally, this study compares the effectiveness of various obesity and metabolic indicators in diagnosing testosterone deficiency, offering valuable insights for clinical application.

## 2 Materials and methods

This cross-sectional study utilized data from the NHANES, which is conducted by the National Center for Health Statistics (NCHS) of the United States. NHANES is a comprehensive survey designed to collect representative information about the health and nutrition of the civilian population of the United States, including demographics, socio-economic status, dietary, and health-related questions. To ensure sample diversity, NHANES employs a stratified, multi-stage sampling method to select participants from across the country. The study protocol was approved by the Research Ethics Review Committee of the National Center for Health Statistics at the Centers for Disease Control and Prevention (CDC), and all NHANES processes were approved by the NCHS Research Ethics Review Board (ERB), besides, participants provided written informed consent. However, as this text involves secondary analysis of data already published in the NHANES database and does not involve patient privacy and safety, it does not require renewed informed consent from participants or ethical approval from the ERB. More detailed information can be obtained from the official NHANES website. (https://www.cdc.gov/nchs/nhanes/irba98.htm)

### 2.1 Selection criteria for study population

In this study, we focus on the population from the NHANES 2011–2016 survey cycle. The initial sample includes 29,902 participants. To identify the population eligible for analyses, the exclusion criteria were as follows: (1) Participants with incomplete data required to calculate CMI (including height, waist circumference, HDL, and TG). (2) Participants with incomplete serum testosterone data. (3) Female participants. (4) Male participants under the age of 18.

**Fig 1:**
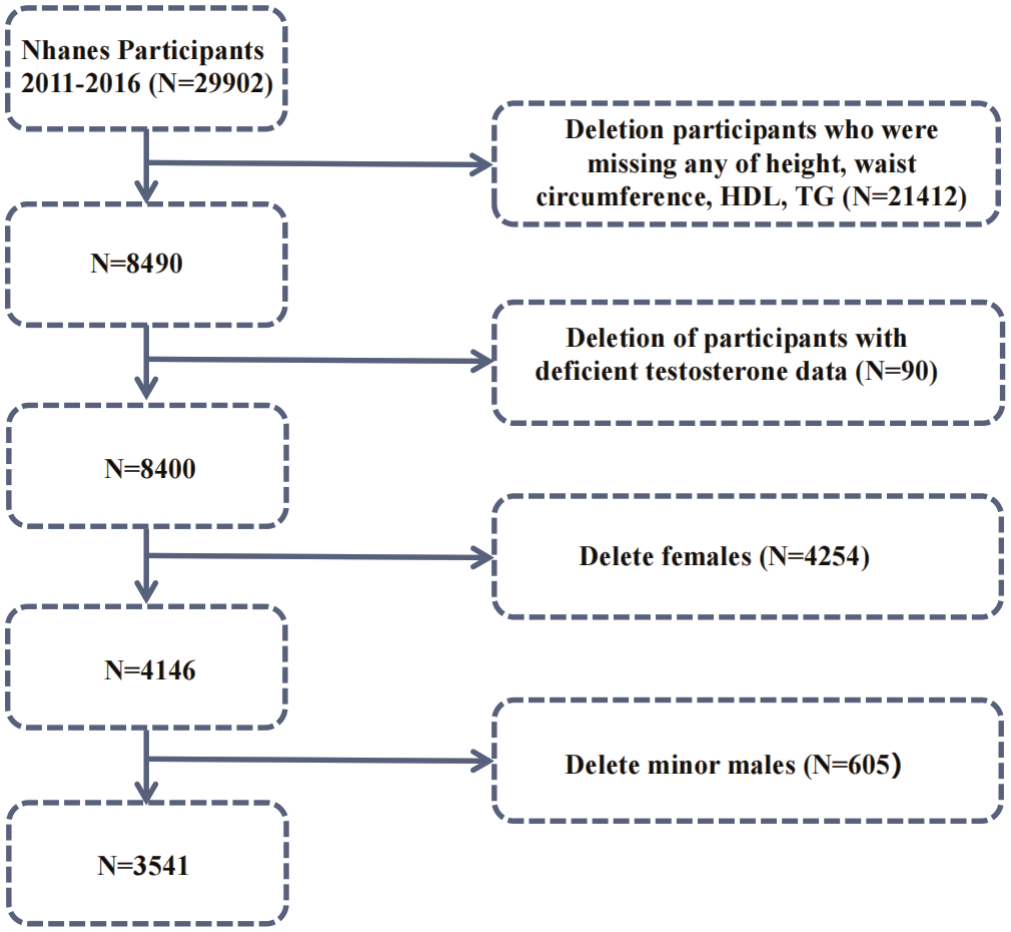
Flow chart of the participants selection process.

Ultimately, 3,541 participants were recruited as the primary study subjects, as shown in Fig 1.

### 2.2 Assessment of CMI and other indicators

CMI is the exposure variable in our study, calculated using the following formula [15]:

$$\text{CMI}=\frac{\text{TG(mg/dl})}{\text{HDL(mg/dl})}\times \frac{\text{waist}(\text{cm})}{\text{height}(\text{cm})}$$

Other formulas for calculating obesity-related indices are as follows [22–26]:

$$\text{BMI}=\frac{\text{weight(kg)}}{\text{height}{\left(\text{m}\right)}^{\text{2}}}$$

$$\text{WWI}=\frac{\text{waist}(\text{cm})}{\sqrt{\text{weight}(\text{kg})}}$$

$${\text{VAI}}_{\left(\text{men}\right)}=\frac{\text{waist}(\text{cm})}{39.68+(1.88\times \text{BMI})}\times \frac{\text{TG}(\text{mg}/\text{dl})}{1.03}\times \frac{1.31}{\text{HDL}(\text{mg}/\text{dl})}$$

$${\text{LAP}}_{\left(\text{men}\right)}=[\text{waist}(\text{cm})-65\text{cm]}\times \text{TG}(\text{mmol}/\text{L)}$$

$$\text{TyG}=\text{Ln}[\frac{\text{TG}(\text{mg}/\text{dl})\times \text{GL(mg/dl)}}{\text{2}}]$$

Meanwhile, this trial divided participants into four groups based on the quartiles of CMI. More specifically, using the first quartile (CMIQ1) as the reference, this included Quartile 1 (CMIQ1≤25th percentile, 0.09–0.71), Quartile 2 (CMIQ2>25th percentile to ≤50th percentile, 0.71–1.22), Quartile 3 (CMIQ3>50th percentile to ≤75th percentile, 1.22–2.25), and Quartile 4 (CMIQ4>75th percentile, 2.25–21.04).

### 2.3 Assessment of serum testosterone level

In this study, total serum testosterone (both free and protein-bound testosterone) and testosterone deficiency were used as the dependent variables, measured using the Isotope Dilution Liquid Chromatography-Tandem Mass Spectrometry (ID-LC-MS/MS) developed by CDC. Testosterone was extracted from samples using liquid-liquid extraction techniques, then analyzed using ID-HPLC/MS/MS and a triple quadrupole mass spectrometer. This process was conducted in positive ion mode, utilizing electrospray ionization to assist in the analysis. Additionally, ^13C-labeled testosterone was used as an internal standard, comparing chromatographic retention times and specific mass-to-charge ratio transitions to identify and quantify testosterone. This measurement method for testosterone has been certified by the CDC Hormone Standardization Program (HoSt) and has been proven over many years to be highly accurate and precise [27]. Meanwhile, the diagnosis of testosterone deficiency is determined based on a total testosterone level of less than 300 ng/dl [28].

### 2.4 Covariates

To identify potential confounders, this study included several covariates that could affect the relationship between serum testosterone and the CMI. These covariates encompass a range of demographic characteristics of the study population, including age, race, marital status, educational level, and income-to-poverty ratio, as well as smoking status, drinking habits, and BMI. Smoking history was determined by whether an individual had smoked more than 100 cigarettes in their lifetime, and drinking history was defined by whether they had ever consumed one 12-ounce beer, one 5-ounce glass of wine, or 1.5 ounces of liquor within the past year. Additionally, the study considered common diseases that might affect metabolic levels, such as diabetes, hypertension, dislipidemia, coronary heart disease, and liver diseases, which were identified through survey questionnaires. Specific questionnaire details can be obtained from the NHANES official website.

### 2.5 Statistical analyses

Following recommendations from NCHS, appropriate weights were applied to each analysis. For continuous variables, one-way analysis of variance was used, while chi-square tests were employed for categorical variables. Smooth curve fitting was utilized to assess the linear relationship between CMI and testosterone. Three different models were used for multivariable regression analysis to test the relationship between CMI and serum testosterone. In Model 1, no covariates were adjusted for. In Model 2, adjustments were made for age and race. In Model 3, adjustments were made for age, marital status, race, income-to-poverty ratio, education level, smoking and drinking habits, BMI, hypertension, diabetes, coronary heart disease, dislipidemia, and liver diseases. Subgroup analyses were also conducted on the factors mentioned in Model 3 to assess the stability and reliability of our study. Finally, receiver operating characteristic (ROC) curves were drawn for multiple obesity prediction indices, and the areas under the curve (AUC) were calculated to compare the specificity and sensitivity of each diagnostic indicator. All results of this study were performed using R (4.2.1) and Empower Stats (4.1).

## 3 Results

### 3.1. Characteristics of participants

We analyzed data from 3,541 participants collected by NHANES from 2011 to 2016 to investigate the relationship between CMI and total serum testosterone. Since both CMI and serum testosterone are continuous variables, we grouped the data based on quartiles of CMI. Significant differences (*P* <0.05) were observed across the four groups in terms of race, age, lipid profile, glucose, height, weight, waist circumference, BMI, WWI, VAI, LAP, TyG, testosterone levels, income-to-poverty ratio, marital status, smoking status, dislipidemia, and hypertension. The Q4 group exhibited higher disease risks, poorer lifestyle habits, and lower androgen levels compared to the other groups, as detailed in Table 1.

**Table 1:** Characteristics of participants.

CMI	Q1	Q2	Q3	Q4	*P* value
**N**	885	885	885	886	
**Age(years)**	44.41±20.03	47.35±18.54	49.87±17.95	48.99±16.15	<0.001
**TC(mg/dl)**	173.51±36.01	180.87±39.02	184.89±40.41	201.26±44.49	<0.001
**TG(mg/dl)**	56.80±17.14	87.53±21.52	123.17±28.81	246.21±130.83	<0.001
**LDL(mg/dl)**	98.86±30.51	112.34±34.15	115.79±36.43	117.18±38.73	<0.001
**HDL(mg/dl)**	63.30±15.65	51.01±9.38	44.44±8.15	37.55±7.35	<0.001
**GLU(mg/dl)**	103.64 ± 26.50	106.95 ± 29.41	112.72 ± 35.80	121.80 ± 46.71	<0.001
**Testosterone(ng/dl)**	544.54±197.44	477.97±186.47	422.82±184.09	365.38±155.25	<0.001
**Height(cm)**	174.24±7.25	174.54±7.52	173.60±7.41	173.71±7.93	0.027
**Waist(cm)**	88.69±12.22	97.97±13.95	104.26±14.98	109.36±15.51	<0.001
**Weight(kg)**	74.64±14.59	83.43±17.60	89.55±20.44	96.22±23.20	<0.001
**BMI**	24.44±4.11	27.53±5.3	29.74±5.87	31.75±6.29	<0.001
**WWI**	10.31±0.82	10.72±0.76	11.04±0.73	11.20±0.69	<0.001
**VAI**	1.22±0.39	2.34±0.43	3.87±0.76	9.71±6.32	<0.001
**LAP**	15.03 ± 8.63	31.13 ± 11.72	52.65 ± 18.73	119.53 ± 69.56	<0.001
**TyG**	8.33 ± 0.59	8.58 ± 0.63	8.73 ± 0.69	8.94 ± 0.71	<0.001
**Income-poverty ratio**	2.48±1.66	2.66±1.68	2.47±1.62	2.44±1.61	0.031
**Marital status(%)**					<0.001
**Married**	47.48%	57.78%	58.13%	62.36%	
**Unmarried**	44.96%	33.85%	31.70%	27.94%	
**Divorced**	7.56%	8.37%	10.18%	9.70%	
**Education(%)**					0.520
**High school graduate and under**	47.61%	44.86%	47.84%	48.04%	
**College and above**	52.39%	55.14%	52.16%	51.96%	
**race(%)**					<0.001
**White**	35.93%	39.66%	38.19%	45.26%	
**Black**	29.38%	21.92%	16.95%	10.05%	
**Others**	34.69%	38.42%	44.86%	44.70%	
**Drinking(%)**					0.437
**Yes**	79.95%	81.57%	83.33%	83.07%	
**No**	20.05%	18.43%	16.67%	16.93%	
**Smoking(%)**					0.038
**Yes**	29.72%	33.90%	39.55%	39.50%	
**No**	70.28%	66.10%	60.45%	60.50%	
**Diabetes(%)**					0.051
**Yes**	14.26%	16.49%	18.51%	20.24%	
**No**	85.74%	83.51%	81.49%	79.76%	
**Dislipidemia(%)**					<0.001
**Yes**	42.78%	45.38%	50.26%	54.22%	
**No**	57.22%	54.62%	49.74%	45.78%	
**Hypertension(%)**					<0.001
**Yes**	25.76%	33.11%	36.84%	42.33%	
**No**	74.24%	66.89%	63.16%	57.67%	
**Liver Diseases(%)**					0.369
**Yes**	1.97%	3.26%	3.20%	2.96%	
**No**	98.03%	96.74%	96.80%	97.04%	
**Coronary heart disease(%)**					0.182
**Yes**	1.60%	2.42%	3.08%	3.08%	
**No**	98.03%	96.74%	96.80%	97.04%	

**Note:** Q = quartile, TC:Total Cholesterol,TG:Triglycerides,LDL:Low-Density Lipoprotein Cholesterol,HDL:High-Density Lipoprotein Cholesterol,GLU:Glucose,CMI:cardiometabolic index, BMI:body mass index, WWI:weight-adjusted-waist index,VAI:visceral adiposity index LAP:lipid accumulation product,TyG:Triglyceride glucose index.

### 3.2. Association between CMI and testosterone

**Fig 2:**
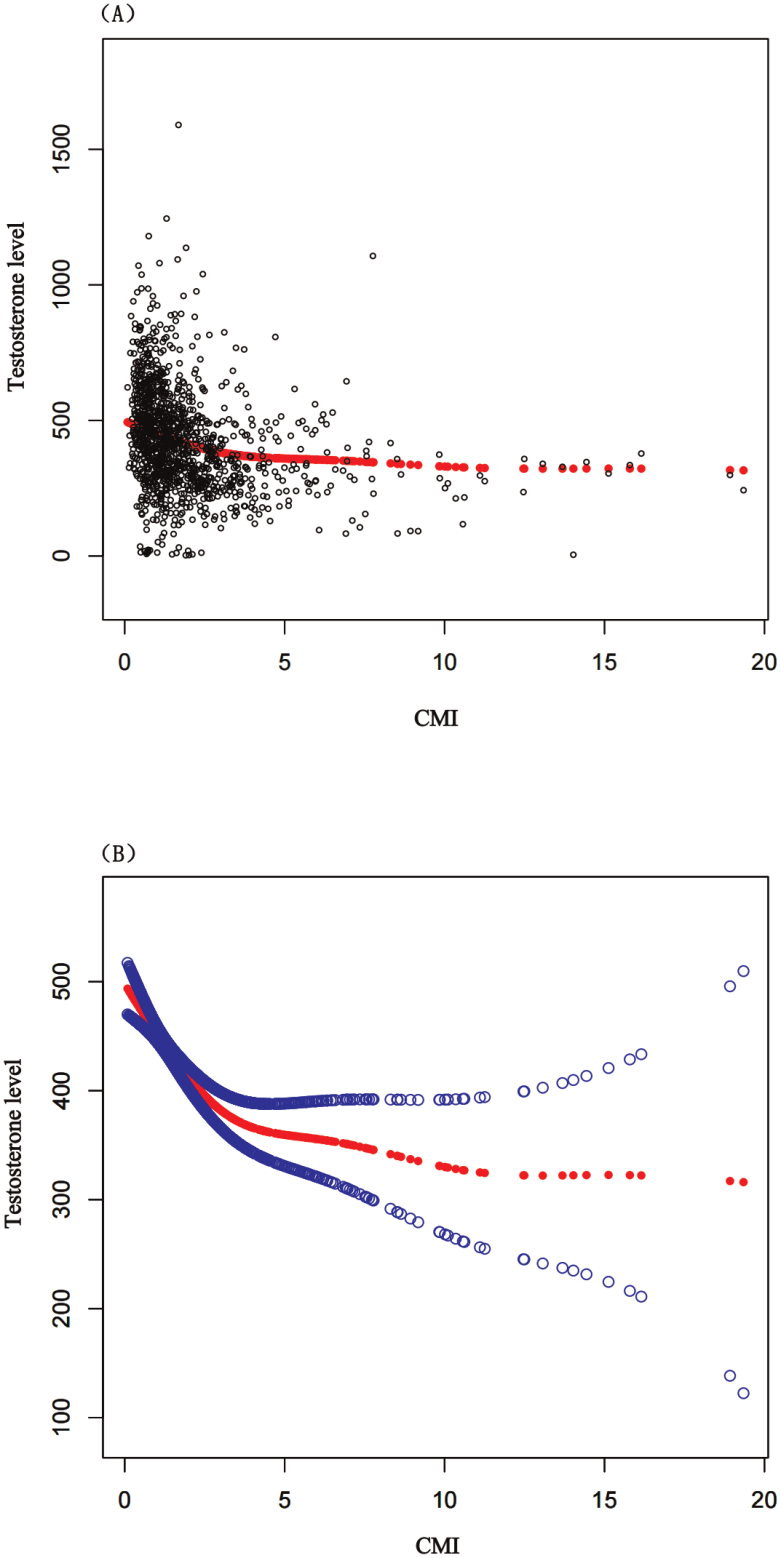
The association between CMI and testosterone level. (A) Each black dot represents a sample from a participant. (B) The red line represents the curve fitting between the independent variable and the dependent variable. The blue line indicates the 95% confidence interval for the fit.

**Fig 3:**
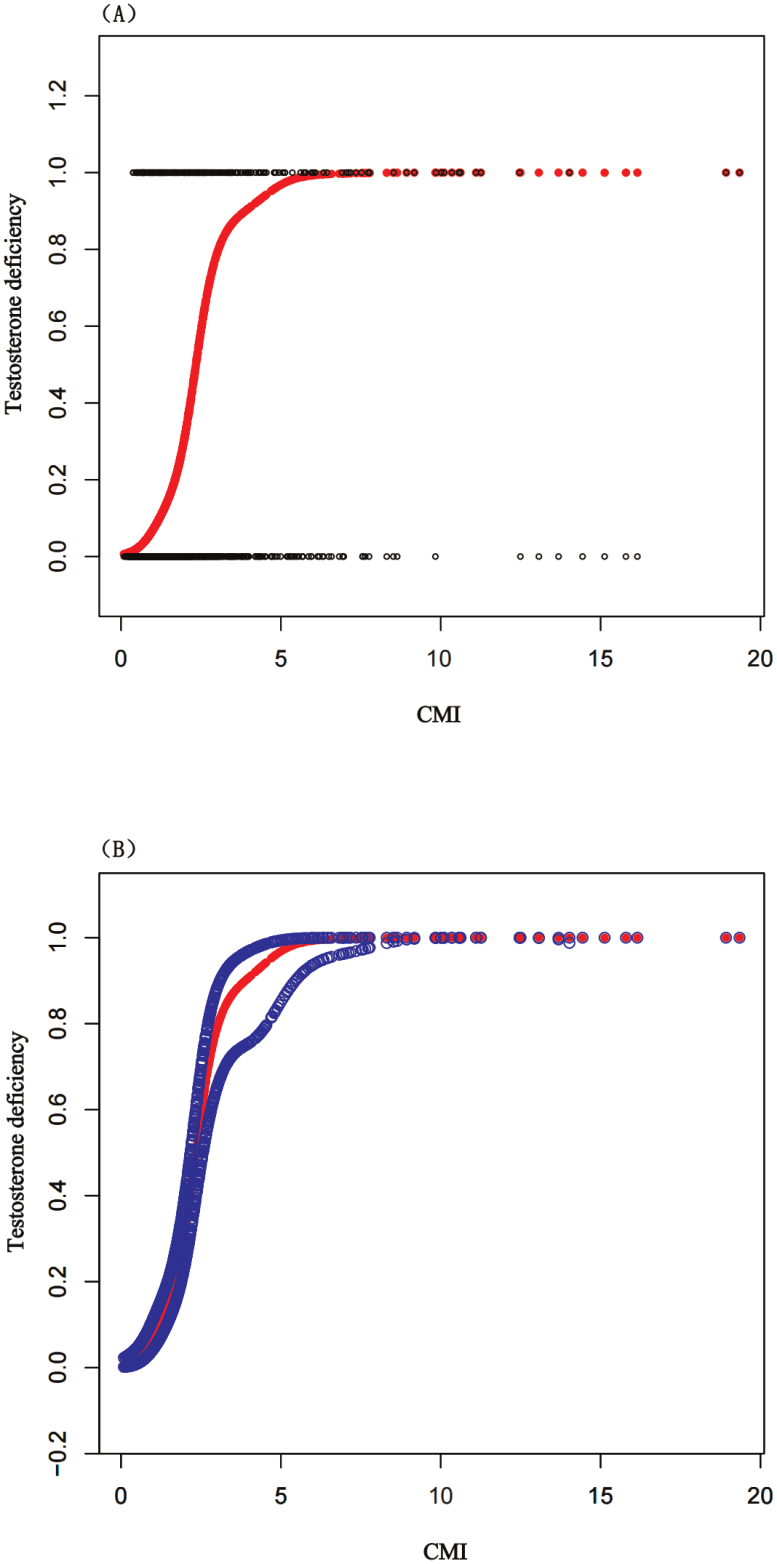
The association between CMI and testosterone deficiency. (A) Each black dot represents a sample from a participant. (B) The red line represents the curve fitting between the independent variable and the dependent variable. The blue line indicates the 95% confidence interval for the fit.

After conducting multivariable regression analyses with three models, we found a negative correlation between the CMI and serum total testosterone levels. In the fully adjusted Model 3, the independent effect was (β = -14.89, 95% CI: -19.97, -9.81), indicating that for every unit increase in CMI, testosterone decreases by 14.89 ng/dl, as shown in Table 2. This negative correlation persisted across quartile groups of CMI (*P* for Trend<0.0001), and was most pronounced in the Q4 group (β = -98.58, 95% CI: -131.81, -65.34), as shown in Table 3. Additionally, we found that for each unit increase in CMI, the incidence of testosterone deficiency increased by 16%, (OR = 1.16, 95% CI: 1.08, 1.25), as details in Table 4. To assess whether the correlation between CMI and testosterone levels was nonlinear, further smooth curve fitting analyses were conducted. The results confirmed that participants with a higher CMI had lower testosterone levels, indicating a negative correlation, as shown in Fig 2. Additionally, participants with a higher CMI were more likely to suffer from testosterone deficiency, showing a positive correlation, as detailed in Fig 3.

**Table 2:** Multivariate logistic regression modelling of CMI and testosterone.

Model 1		Model 2		Model 3	
β(95% CI)	*P* value	β(95% CI)	*P* value	β(95% CI)	*P* value
-24.29 (-27.27, -21.32)	<0.0001	-23.57 (-26.53, -20.61)	<0.0001	-14.89 (-19.97, -9.81)	<0.0001

**Note:** Model 1:unadjusted; Model 2: adjust for age, race; Model 3: adjust for age, race, marital status, Income-poverty ratio, BMI, education, smoking, drinking, Coronary heart disease, Diabetes, Hypertension, Dislipidemia, Liver diseases.

**Table 3:** Multivariate regression analysis of CMI groups and testosterone.

	Model 1		Model 2		Model 3	
CMI Groups	β(95% CI)	*P* value	β(95% CI)	*P* value	β(95% CI)	*P* value
**Q1:0.09–0.71**	Ref	-	Ref	-	Ref	-
**Q2:0.71–1.22**	-66.57 (-83.48, -49.66)	<0.0001	-63.12 (-79.88, -46.35)	<0.0001	-8.48 (-38.65, 21.68)	0.5816
**Q3:1.22–2.25**	-121.72 (-138.62, -104.81)	<0.0001	-115.47 (-132.39, -98.55)	<0.0001	-38.03 (-68.70, -7.36)	0.0152
**Q4:2.25–21.04**	-179.16 (-196.06, -162.25)	<0.0001	-174.88 (-191.90, -157.86)	<0.0001	-98.58 (-131.81, -65.34)	<0.0001
***P* for trend**	-56.43 (-61.88, -50.99)	<0.0001	-55.02 (-60.48, -49.55)	<0.0001	-35.18 (-45.14, -25.23)	<0.0001

**Note:** Model 1:unadjusted; Model 2: adjust for age, race; Model 3: adjust for age, race, marital status, Income-poverty ratio, BMI, education, smoking, drinking, Coronary heart disease, Diabetes, Hypertension, Dislipidemia, Liver diseases.

**Table 4:** Multivariate regression analysis of CMI and testosterone deficiency.

Model 1		Model 2		Model 3	
OR (95%CI)	*P* value	OR (95%CI)	*P* value	OR (95%CI)	*P* value
1.25 (1.20, 1.30)	<0.0001	1.26 (1.21, 1.31)	<0.0001	1.16 (1.08, 1.25)	<0.0001

**Note:** Model 1:unadjusted; Model 2: adjust for age, race; Model 3: adjust for age, race, marital status, Income-poverty ratio, BMI, education, smoking, drinking, Coronary heart disease, Diabetes, Hypertension, Dislipidemia, Liver diseases.

### 3.3. Results of subgroup analyses

To ascertain the stability of the relationship between the CMI and serum total testosterone levels, subgroup analyses were performed. Results indicated that the negative correlation between CMI and testosterone remained stable across most subgroups, except for participants who had a BMI below 18.5, were divorced, of Black race, or diagnosed with diabetes, liver diseases, or coronary heart disease. Additionally, interaction tests showed that the relationship between CMI and testosterone levels was not associated with race, age, lifestyle habits, BMI, WWI, marital status, hypertension, dislipidemia, or coronary artery disease (*P* for interaction>0.05), but may be related to liver diseases (*P* for interaction<0.05), as detailed in Table 5.

**Table 5:** Subgroup analyses of the association between CMI and testosterone level.

Subgroup	β(95% CI)	*P* for interaction
**Age**		0.208
**<45**	-19.30 (-27.65, -10.96)	
**>45**	-12.90 (-19.07, -6.73)	
**Drinking**		0.923
**Yes**	-14.95 (-20.49, -9.40)	
**No**	-14.33 (-25.98, -2.68)	
**Smoking**		0.257
**Yes**	-12.00 (-19.11, -4.89)	
**No**	-17.42 (-24.23, -10.62)	
**BMI**		0.146
**<18.5**	-197.23 (-473.02, 78.56)	
**18.5–24.9**	-32.42 (-49.70, -15.14)	
**25–29.9**	-15.59 (-23.87, -7.30)	
**≥30**	-14.67 (-21.46, -7.87)	
**Race**		0.135
**White**	-15.27 (-21.97, -8.57)	
**Black**	-1.44 (-16.51, 13.63)	
**Others**	-18.07 (-26.33, -9.82)	
**Marital status**		0.147
**Married**	-19.95 (-27.40, -12.50)	
**Unmarried**	-10.52 (-17.25, -3.79)	
**Divorced**	-19.70 (-44.10, 4.69)	
**Education**		0.951
**High school graduate and under**	-14.94 (-21.90, -7.97)	
**College and above**	-14.64 (-21.60, -7.68)	
**Coronary heart disease**		0.998
**Yes**	-14.82 (-34.01, 4.37)	
**No**	-14.85 (-20.09, -9.60)	
**Diabetes**		0.135
**Yes**	-8.87 (-18.23, 0.49)	
**No**	-17.03 (-22.91, -11.15)	
**Dislipidemia**		0.570
**Yes**	-13.48 (-20.31, -6.65)	
**No**	-16.28 (-23.54, -9.01)	
**Hypertension**		0.149
**Yes**	-10.19 (-16.82, -3.56)	
**No**	-20.66 (-28.04, -13.27)	
**Liver Diseases**		0.036
**Yes**	3.73 (-14.53, 21.98)	
**No**	-16.30 (-21.56, -11.03)	

### 3.4 Evaluation of diagnostic tests using ROC curves

**Fig 4:**
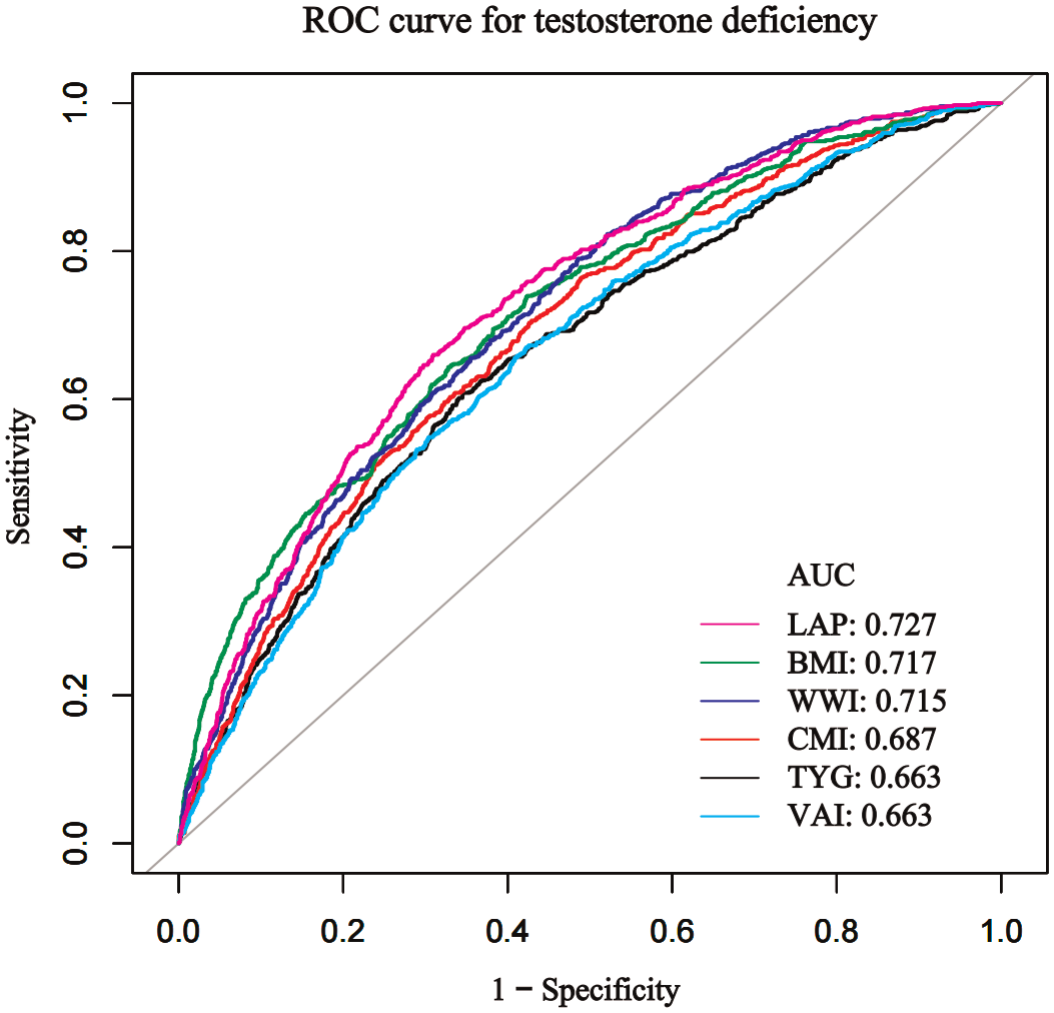
ROC curve analysis for different indicators in predicting testosterone deficiency.

ROC analysis, as shown in Fig 4 and Table 6, revealed that the AUCs (95% CI) for LAP, BMI, WWI, CMI, VAI and TyG were 0.73 (0.71–0.75), 0.72 (0.70–0.74), 0.71 (0.69–0.74), 0.69 (0.67–0.71), 0.66 (0.64–0.69), and 0.66 (0.64–0.69) respectively. LAP demonstrated the highest predictive capability, while VAI and TyG were the lowest.

**Table 6:** Comparison of different indicators for predicting testosterone deficiency.

Test	Best threshold	Specificity	AUC	95%CI low	95%CI up	Sensitivity	Accuracy	Postive-pv	Negative-pv
**CMI**	1.28	0.57	0.69	0.67	0.71	0.70	0.60	0.31	0.88
**BMI**	27.66	0.58	0.72	0.70	0.74	0.74	0.61	0.31	0.90
**WWI**	10.73	0.51	0.71	0.69	0.74	0.79	0.57	0.29	0.91
**VAI**	3.11	0.58	0.66	0.64	0.69	0.67	0.60	0.29	0.87
**LAP**	50.86	0.69	0.73	0.71	0.75	0.66	0.68	0.35	0.89
**TyG**	8.76	0.65	0.66	0.64	0.69	0.61	0.65	0.30	0.86

**Note:** CMI:cardiometabolic index, BMI:body mass index, WWI:weight-adjusted-waist index,VAI:visceral adiposity index LAP:lipid accumulation product,TyG:Triglyceride and glucose index

## 4 Discussion

In this cross-sectional study, we discovered a negative correlation between the CMI and testosterone levels in adult males, with higher CMI being associated with lower testosterone levels and an increased risk of testosterone deficiency. Smooth curve fitting analysis revealed a negative nonlinear relationship between CMI and testosterone levels, as well as between CMI and testosterone deficiency. As an index that integrates lipid indicators and somatic measurements, CMI can predict the relationship between obesity, metabolic conditions, and other diseases. Although no studies have yet explored the direct relationship between CMI and testosterone, indirect evidence can be found in research linking lipid profiles, body measurements, and testosterone levels. Previous indicators used to estimate testosterone levels, such as BMI, WWI, VAI, LAP, and TyG, share similar calculation elements with CMI, such as height, triglycerides, and waist circumference, indirectly demonstrating CMI’s ability to assess testosterone levels [18–21]. Additionally, the degree of obesity and metabolic conditions reflected by CMI are closely related to testosterone levels. Obese individuals have adipose tissue rich in active aromatase, which can convert testosterone into estradiol, reducing body’s testosterone levels [29]. Elevated circulating triglycerides can promote adipocyte hypertrophy and hyperfunction [30], leading to the secretion of inflammatory factors such as leptin, tumor necrosis factor (TNF), and interleukin-6 (IL-6), which decrease the secrete of testosterone, ultimately resulting in reduced testosterone secretion [31]. These pieces of evidence indirectly corroborates the relationship between CMI and testosterone.

Additionally, we observed that the negative correlation between CMI and testosterone levels remained stable in most subgroups, except for participants with a BMI under 18.5, those who are divorced, of Black race, or diagnosed with diabetes, liver disease, or coronary heart disease. This indicates that in most cases, the relationship between CMI and testosterone levels is stable. The instability in the relationship in certain groups may be due to several factors. For underweight patients (BMI<18.5), malnutrition or other causes may lead to insufficient cholesterol and protein necessary for hormone synthesis [32], potentially affecting the link between CMI and testosterone levels, although overall, BMI does not impact the relationship between CMI and testosterone levels.For divorced individuals, the impact of CMI on testosterone levels may not be apparent, possibly due to the intense sorrow, anxiety, and depression triggered by divorce. Long-term psychological stress can cause fluctuations in cortisol secretion, thereby affecting testosterone secretion [33, 34], making the connection between CMI and testosterone levels unstable. Nevertheless, it should be noted that, in general, marital status does not significantly influence the relationship between CMI and testosterone levels. Additionally, the association between CMI and testosterone levels is not strongly established among individuals of Black race, potentially due to higher testosterone levels and greater physiological variability within this group. [35]. Additionally, for participants with diabetes or coronary heart disease, the relationship between CMI and testosterone levels is not stable, possibly because these patients are already in a state of metabolic disorder and chronic inflammation [36–38], which can influence the relationship between CMI and testosterone levels, thus reducing the stability of the link between CMI and testosterone in these patients. Finally, among participants with liver disease, the relationship between CMI and testosterone levels was also found to be unstable, with a noticeable interaction effect. This suggests that the presence or absence of liver disease might affect the relationship between CMI and testosterone levels, likely because the liver is involved in the metabolism of testosterone [39, 40].

It has been established that previous researchers have evaluated the feasibility of using indicators such as BMI, WWI, VAI, LAP, and TyG to predict testosterone levels and testosterone deficiency. Building on their findings, our study further confirms that the CMI can also be used to assess testosterone deficiency and testosterone levels [18–21]. However, previous studies have not conclusively identified which obesity and metabolic index is most effective for predicting testosterone deficiency. To bridge this knowledge gap, we performed a ROC analysis to assess the predictive efficacy of various indicators for testosterone deficiency. The results revealed that LAP has the highest effectiveness in predicting testosterone deficiency, followed by BMI, WWI, CMI, VAI, and TyG. However, BMI does not distinguish between muscle and fat, nor does it consider the distribution of body fat, making it less specific in its measurements [41]. WWI focuses mainly on waist circumference, without considering the different health risks associated with the same waist size at varying heights, we do not regard it as a qualified predictive indicator either [42]. Moreover, both BMI and WWI fail to reflect the role of lipid metabolism in diseases. Therefore, we do not consider the contributions of BMI and WWI in evaluations, including them in the ROC analysis solely for the sake of research completeness. It is noteworthy that our research suggests LAP might be the best predictive indicator for assessing testosterone deficiency, as its AUC value is significantly higher than other indicators. However, when men have a waist circumference less than 65 cm, the LAP value becomes negative (below zero), which complicates data processing. Moreover, LAP does not take into account the impact of height differences on waist circumference, which may limit its applicability [43]. Additionally, VAI and TyG showed the least effectiveness in predicting testosterone deficiency [20], weaker than CMI and other indicators, making them less preferable options for assessing testosterone deficiency. In contrast, CMI takes into account height, waist circumference, and the metabolic profile, combining their strengths and minimizing their weaknesses, thereby provides preferable clinical applicability and predictive value. Nevertheless, we must acknowledge that our results indicate LAP holds the highest diagnostic value for testosterone deficiency, followed by CMI, then VAI and TyG. But we also believe that while LAP is not conveniently calculated, CMI can fully serve as a substitute indicator for LAP. Last, it’s important to note that, this analysis pertains only to the effectiveness of these indicators in diagnosing testosterone deficiency, and further analyses is needed to determine which indicator is more accurate in predicting testosterone levels.

Overall, this study has several strengths. Our research is the first to analyze the relationship between the CMI and testosterone levels, yielding convincing results. Additionally, we compared the efficacy of the CMI in predicting testosterone deficiency with various obesity and metabolic indicators, concluding that LAP has the strongest predictive power for testosterone deficiency, followed by CMI, VAI, and TyG. These findings can aid clinicians in comparing and selecting these indicators. Moreover, the study highlights the need for both the public and medical professionals to be aware of the impacts of metabolic status and obesity on testosterone levels.

However, this study still has some limitations. Firstly, there are many potential factors affecting CMI and testosterone levels, and we were unable to adjust for all potential confounders, so the conclusions of this study should be verified through further research. Secondly, this trial only collected data from adult males aged 18 and above, which ignores the impact of CMI levels on testosterone levels in women and minors, which is worth further exploration in the future. Additionally, although cross-sectional studies are useful for preliminary exploratory research and hypotheses, they are limited in that they cannot provide causal evidence. Future research should consider using more longitudinal designs or experimental methods to deepen understanding of the research question. Lastly, due to significant differences in genetic backgrounds, lifestyles, medical standards, and socio-economic conditions among different countries and regions, the findings of this study may not be entirely applicable to other countries. Therefore, caution is needed when applying these results to other parts of the world, taking into account the unique environmental and demographic characteristics of those regions. Future studies should expand the sample range to include more countries and cultures to enhance the generalizability and applicability of the research findings.

## 5 Conclusion

Our study demonstrates that higher levels of CMI are associated with lower testosterone levels and a higher risk of testosterone deficiency, and this negative correlation remained unchanged adjusting for the various covariates. Additionally, we compared the predictive values of several indices in diagnosing testosterone deficiency and found that LAP has a higher predictive value than CMI, while CMI’s predictive value is higher than VAI and TyG. In summary, the CMI offers a way to monitor serum testosterone levels and suggests that patients with testosterone deficiency manage their obesity and lipid levels. These findings have significant implications for clinical decision-making and preventative measures.

## Supporting information

S1 Data Original data information for participants. (XLSX)

## Abbreviations

BMI: Body Mass Index
CMI: Cardiometabolic Index
HDL: High-Density Lipoprotein
LAP: Lipid Accumulation Product
NCHS: National Center for Health Statistics
NHANES: National Health and Nutrition Examination Survey
TG: Triglyceride
TyG: Triglyceride glucose index
VAI: Visceral Adiposity Index
WWI: Weight Adjusted Waist Index
